# Immediate Irradiation Induced Cerebral Water and Hemodynamic Response in Whole Brain Radiotherapy

**DOI:** 10.1007/s10439-024-03663-1

**Published:** 2024-12-04

**Authors:** Heli Miettinen, Jesse Lohela, Sadegh Moradi, Kalle Inget, Juha Nikkinen, Teemu Myllylä, Sakari S. Karhula, Vesa Korhonen

**Affiliations:** 1 Department of Diagnostic Radiology, Oulu University Hospital, Oulu, Finland; 2 Department of Oncology and Radiotherapy, Oulu University Hospital, Oulu, Finland; 3 Research Unit of Health Sciences and Technology, University of Oulu, Oulu, Finland; 4 Optoelectronics and Measurement Techniques Unit, University of Oulu, Oulu, Finland; 5 Medical Research Center, Oulu, Finland

**Keywords:** Brain, Radiotherapy, Functional near-infrared spectroscopy, Hemodynamics, Cerebrospinal fluid, Blood–brain barrier

## Abstract

**Purpose:**

Effects of clinical radiotherapy are often studied between or after irradiations. The current study’s aim was to monitor an immediate irradiation response in cerebral water and hemodynamics in patients treated with whole brain radiotherapy (WBRT) and to assess the response’s individuality.

**Methods:**

We used functional near-infrared spectroscopy (fNIRS) to monitor changes in cerebral water, oxyhemoglobin (HbO), and deoxyhemoglobin (HbR) during the irradiation of 31 patients (age 69.3 ± 12.5 years, 16 females) receiving WBRT. The radiation dose delivered to a patient during a single measurement was 4 Gy (total dose of 20 Gy in five fractions) for most patients and 3 Gy (total dose of 30 Gy in ten fractions) for three patients.

**Results:**

106 patient recordings were analyzed. They showed an immediate irradiation induced increase in HbO and HbR, and decrease in cerebral water content (*P* < .001) as soon as 5 s after the start of irradiation. The radiation dose, age, and gender affected recorded signals. A smaller dose resulted in a steeper change in HbR (*P* < .01), but larger total change in HbO (*P* < .01). Younger age was associated with a more significant decrease in the water signal (*P* < .05). In contrast, female gender was associated with a greater total increase in HbO (*P* < .01) and HbR (*P* < .001) signals.

**Conclusion:**

There is an immediate cerebral water and hemodynamic response to irradiation and this response shows dependency on the radiation dose, age, and gender. Better understanding about the immediate radiation response may help improve the patient outcome in clinical radiotherapy.

**Supplementary Information:**

The online version contains supplementary material available at 10.1007/s10439-024-03663-1.

## Introduction

The most common type of neurological complications of systemic cancer in adults are brain metastases. They can occur with any primary malignant tumor, but the most common sources are lung cancer, breast cancer, and melanoma [1]. While the exact portion of cancer patients that develop brain metastases is uncertain, it is estimated to be around 20% [1–3]. The prognosis of the patients has typically been poor but has improved recently, thanks to improved treatment methods. One frequently utilized therapeutic modality is whole brain radiotherapy (WBRT), which can be administered either independently or as an adjunctive therapy. When used alone, it is typically employed as a palliative treatment for patients with multiple brain metastases [2, 3]. WBRT involves subjecting the entire brain to irradiation, providing an opportunity to investigate the effects of irradiation on brain tissue. To our knowledge, this approach has not been applied during irradiation, which was the primary focus of the present study. A comprehensive understanding of the impact of radiation on the brain is crucial for enhancing brain radiotherapy methods and improving patient outcomes.

Cancer is associated with increased tissue hydration, which has even been concluded to be a major factor in carcinogenesis [4–6]. The brain is not an exception here, as vasogenic edema is almost always present with brain metastases [7]. Peritumoral edema in the brain is related to neurological symptoms and morbidity of the patients even more strongly than the metastases themselves [7–10]. Regrettably, edema is also associated with radiotherapy treatment for brain metastases. WBRT is known to induce cerebral edema, probably due to irradiation-induced blood–brain barrier (BBB) disruption (BBBD). The study of irradiation-induced edema has primarily focused on preclinical models exposed to high radiation doses, with recorded onset times varying from 24 h to 90 days [11–13]. To the best of our knowledge, the immediate impact of radiotherapy on brain edema has not been investigated, potentially attributed to the absence of suitable techniques. However, gaining insights into the immediate irradiation response could contribute to a better understanding and mitigation of the adverse effects associated with radiotherapy.

As irradiation-induced edema is associated with BBBD [11], measurement of brain water content could also provide information about the condition of BBB. The BBB serves as the primary site for blood-central nervous system exchange, tightly regulating the flow of molecules to the brain. This regulation poses a challenge for drug delivery to the brain, complicating brain cancer chemotherapy [14]. The precise time frame and mechanism of irradiation-induced BBBD remains unclear. Understanding these aspects of BBBD could help to ensure the timely and effective administration of chemotherapy and other drugs to the brain.

Functional near-infrared spectroscopy (fNIRS) is an optical neuroimaging technique widely used to monitor changes in cerebral blood oxygenation and tissue hemodynamics. Recent work by Myllylä et al. [15] demonstrated the feasibility of fNIRS in monitoring the immediate response to WBRT in vivo, with a subsequent study unveiling a radiotherapy-induced increase in total hemoglobin [16]. Furthermore, fNIRS has also exhibited potential in measuring fluctuations in cerebral water content [17] and real-time monitoring of BBBD [18]. Despite this, the application of fNIRS in measuring acute cerebral water content changes and real-time monitoring of BBBD response to WBRT is underexplored. In this study, we aimed to bridge these gaps by using the same fNIRS method as Myllylä et al. [16] to monitor immediate changes in both cerebral water content and hemodynamics in response to WBRT. We hypothesized that there would be an immediate response to irradiation in both cases, and that response would depend on the radiation dose and patient parameters, including age and gender. This approach seeks to contribute to a deeper understanding of the immediate impact of radiotherapy on brain physiology, potentially providing insights for optimizing radiotherapy and improving patient care.

## Materials and Methods

The fNIRS device used in this study utilizes a frequency coding technique, and the raw fNIRS signal was sampled at a rate of 800 Hz. The same arrangements as in a previous study [16] were made to ensure the radiotherapy compatibility of fNIRS measurements. These included bringing only the optical fibers for illumination and detection into the radiotherapy chamber by cable entry, while the fNIRS device was placed behind the maze barrier in the radiotherapy room. Near-infrared light was produced at wavelengths 690, 810, 830, and 980 nm by high-power LEDs manufactured by Roithner. Two fNIRS optodes were attached to the subject’s forehead at the source-detector distance of 2.7 to 3.6 cm to reach the brain cortex [19].

Radiotherapy measurements were acquired from 32 patients, with 31 individuals (mean age: 69.3 ± 12.5 years; 16 females) included for analysis after excluding instances of noisy signals. Written informed consent was obtained from each patient in accordance with the Declaration of Helsinki. This study was also approved by the Ethical Committee of Medical Research in the Northern Ostrobothnia District of Finland (237/2018) and was conducted in accordance with the declaration of Helsinki and GDPR regulations. All the patients had WBRT treatment. Most patients received a total dose of 20 Gy in five daily fractions of 4 Gy, and three patients received a total dose of 30 Gy in ten daily fractions of 3 Gy. Each fraction had a combination of right and left fields (90°/270°) irradiated one at a time. The order of fields varied from patient to patient. All treatments were delivered at the dose rate of 600 MU/min. The forward-intensity modulated radiation (FIMRT) technique was used with 27 patients. When the FIMRT technique was used, the patient was irradiated for an average of 18 s after which the radiation field was modified, and additional short irradiation was delivered, which took 6 s on average. In comparison, patients without FIMRT had in average 20 s of irradiation at once. FIMRT was used with both fields of a fraction, one of them or not at all. Each irradiation was delivered externally with a medical linear accelerator (TrueBeam, TrueBeam Edge or iX, Varian Medical Systems). Patient positioning and fiber locations are shown in detail previously in the study by Myllylä et al. [16].

Measurements with a signal-to-noise ratio (SNR) less than one and channels with plausible technical artefacts within the area of interest were rejected from analysis. Examples of types of rejected signals are shown in Online Resource 1. SNR was defined as a mean-to-standard deviation ratio from 15 to 5 s before the first irradiation period. 106 measurements were analyzed after rejections and only one channel from each measurement was analyzed. Raw measured fNIRS signals were converted to represent temporal changes in oxyhemoglobin (HbO), deoxyhemoglobin (HbR), and cerebral water content using modified Beer-Lambert law [20]. Wavelengths of 690, 830, and 980 nm were used in the analysis performed, to quantify the concentrations of all HbO, HbR and water [17]. The total hemoglobin (HbT) signal was calculated as a sum of HbO and HbR signals. All analyses were done in MATLAB R2022b.

Further data processes were made after filtering the signal to low frequency (below 0.1 Hz) to remove the effects of heartbeat and breathing. Signal from 10 s prior to the irradiation to up to nine post irradiation was used. These sections were normalized by setting the corresponding recorded value at the start of irradiation to zero and maximum range of any of the signals during any sections to 100%. This was done to make different measurements comparable, as our NIRS device can only measure relative concentration instead of the absolute one.

Statistical analysis was done in MATLAB R2022b to a filtered but not normalized signal. The sign test was used for statistical analysis when comparing different time points of the same group of signals, and the Wilcoxon rank sum test was used when comparing separate groups, e.g., different radiation doses. *P* values below 0.05 were determined to be significant. Statistical analysis was done to compare measured signal amplitude between the start of irradiation and at 5, 10, 15, 20, and 25 s after it had started for all measurements, and to compare the difference in the change of signal amplitude during the same time windows between two groups of signals. The study compared signal differences between groups to assess whether variations occurred in the response during the first and second irradiation fields of a single treatment fraction, or if the location of the used channel on the same or opposite side of the patient’s head as the incoming radiation led to discrepancies in the recorded signal. The difference was also assessed between groups of patients divided by the delivered dose, age, or gender.

## Results

**Fig. 1:**
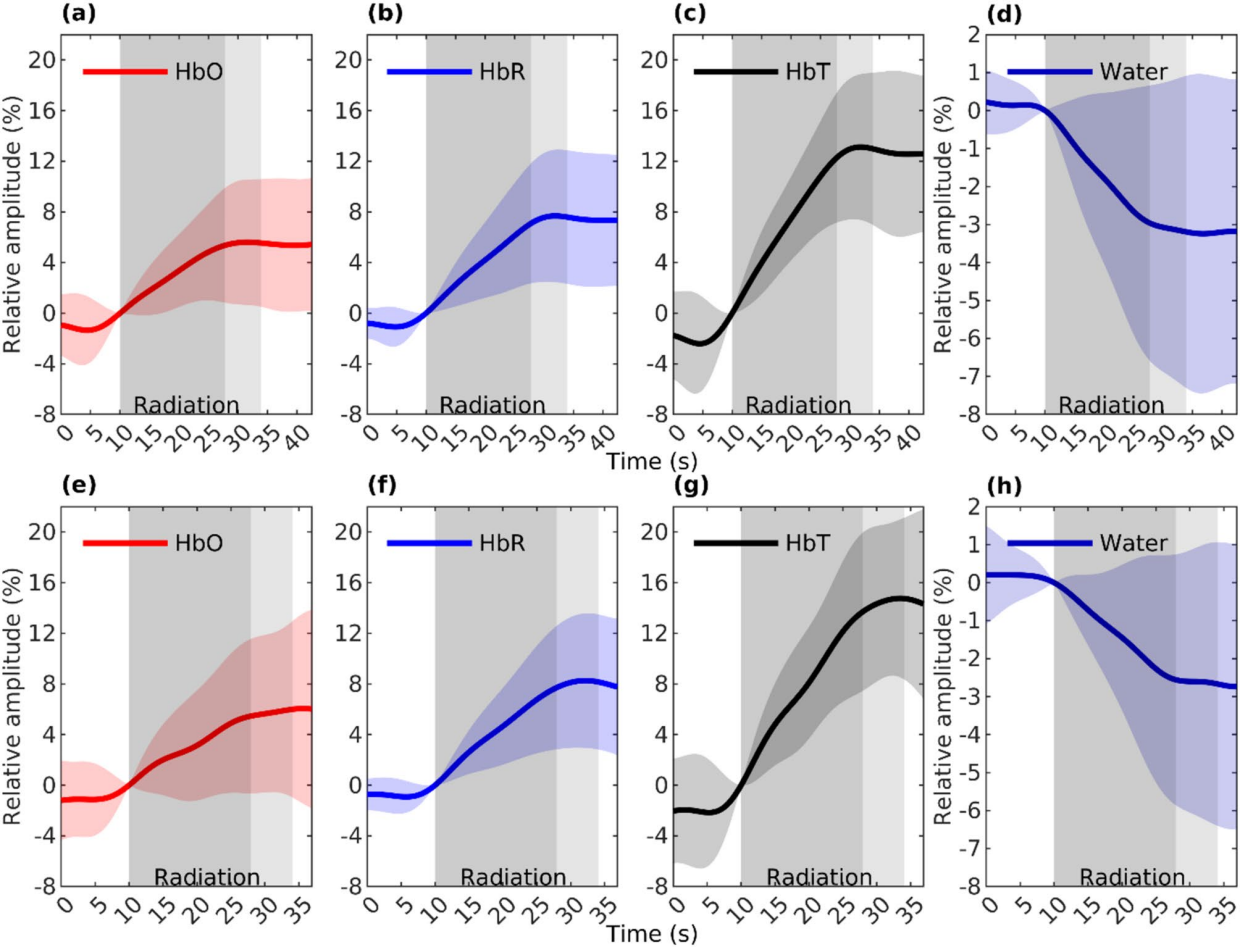
The figure presents the low-pass filtered average (with std) responses of HbO (**a**, **e**), HbR (**b**, **f**), HbT (**c**, **g**), and water (**d**, **h**) to irradiation during the first (**a**–**d**) and the second (**e**–**h**) fields of the treatment fraction, incorporating all *n* = 106 measurements. The dark gray shading illustrates the average duration of irradiation for all patients. The subsequent light gray area indicates a timeframe in which the irradiation field is slightly modified due to the FIMRT technique, which was not administered to all patients. Throughout both irradiations, HbT = HbO + HbR increases, while water decreases linearly

**Fig. 2:**
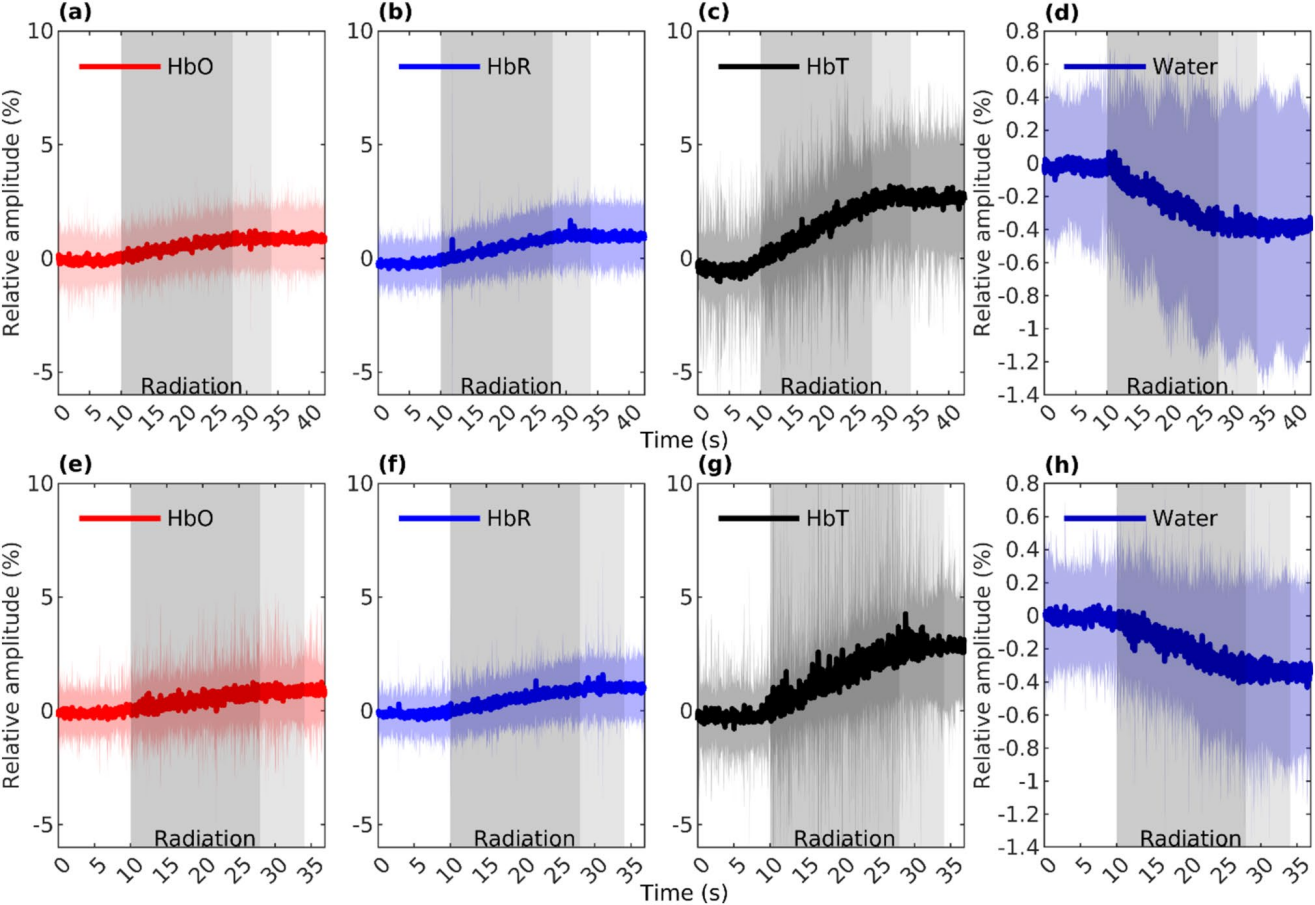
The figure shows full-band average (*n* = 106) responses of HbO (**a**, **e**), HbR (**b**, **f**), HbT (**c**, **g**), and water (**d**, **h**) to irradiation during the first (**a**–**d**) and the second (**e**–**h**) fields of the treatment fraction. The full-band response shows an increase in HbO, HbR, and HbT signals and a decrease in the water signal, highlighting the simultaneous nature of the response with the irradiation. The dark gray segment shows the average duration of the irradiation that all patients had. The following light gray indicates a time frame in which the irradiation field is slightly modified due to the FIMRT technique

**Fig. 3:**
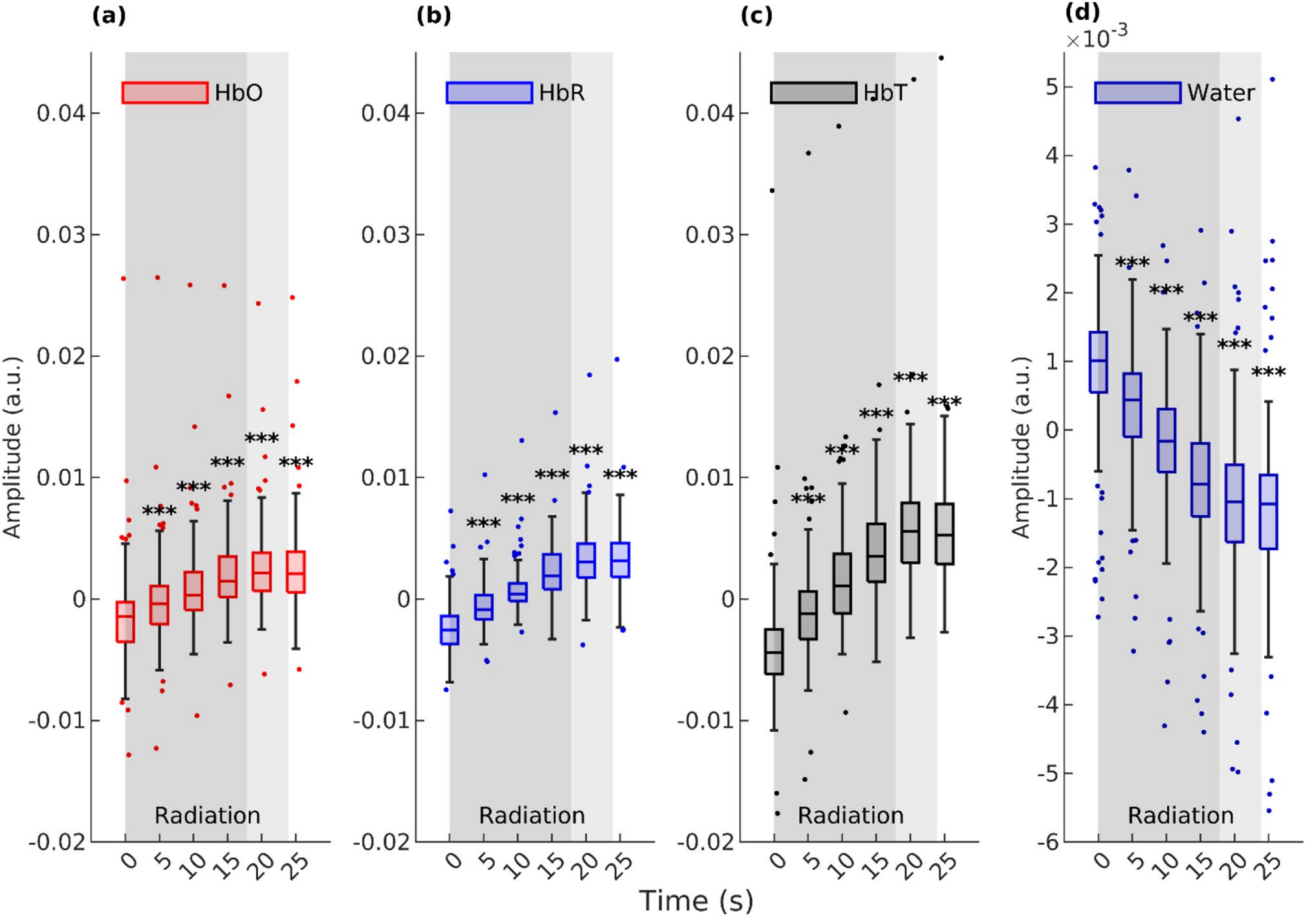
Box plot shows a recorded change in amplitude in all measurements (*n* = 106) at different time points (0 s being the start point of irradiation) for HbO (**a**), HbR (**b**), HbT (**c**), and water (**d**). The dark gray segment shows the average duration of the irradiation that all patients had. The following light gray segment indicates FIMRT. Significant *P* values for the difference between 0 s and a time point in question were calculated from low-pass filtered signals and are shown with asterisks (*** is *P* < .001). There is a statistically significant difference between the start of irradiation and each time point after the start of irradiation for all signals

Our results show a strong linear response in average hemodynamic and cerebral water signals to irradiation. The phenomenon is observable with low-pass filtering (see Fig. 1) and at a full band (see Fig. 2). Statistical analysis revealed this response to be statistically significant (*P* < .001) in the VLF band (see Fig. 3). Between the first and second fields of irradiation in a measurement, no significant difference in responses was detected, nor was between responses from recordings from a channel on the same or opposite side of the patient’s head as the direction of incoming irradiation (*P* > .05).

**Fig. 4:**
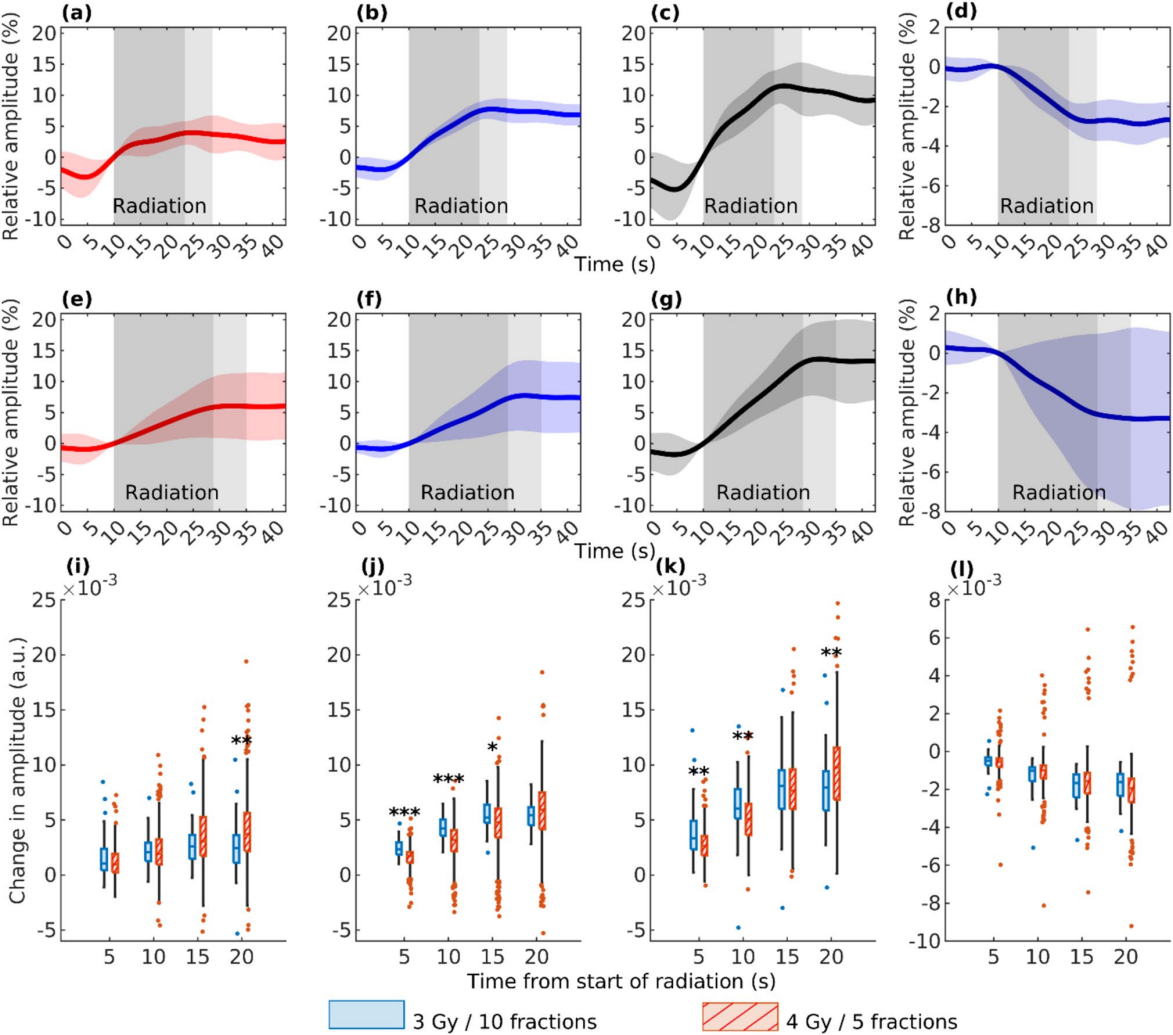
The figure depicts the low-pass filtered average responses of HbO (**a**, **e**), HbR (**b**, **f**), HbT (**c**, **g**), and water (**d**, **h**) to irradiation in patients treated with ten fractions of a 3 Gy dose (**a**–**d**) (*n* = 19) and with five fractions of a 4 Gy dose (**e**–**h**) (n = 87). The dark gray shading indicates the average irradiation duration, which was shorter for the lower dose due to a consistent dose rate. Light gray denotes the inclusion of FIMRT, not administered to all patients. Overall, the responses appear similar for both dose groups. Additionally, changes in the measured amplitude of low-pass filtered HbO (**i**), HbR (**j**), HbT (**k**), and water (**l**) signals from the start of irradiation to 5, 10, 15, and 20 s after initiation are displayed for the two dosages, with the 3 Gy dose in blue and the 4 Gy dose in red. Significant differences in amplitude change are indicated by asterisks (*P < .05, **P < .01, ***P < .001). HbR shows a statistically significant difference in amplitude change near the start of irradiation between the dosages, with the 3 Gy dose exhibiting a steeper change. However, the total change in HbR shows no significant difference. In the HbO signal, a significant difference is observed in the total change

The results show a difference in a low-pass filtered hemodynamic response to two different radiation doses (see Fig. 4). Even though doses were delivered at the same dose rate, the HbR signal response to 3 Gy dose is steeper than the response to 4 Gy dose resulting in the same total response. In the HbO signal, no difference in the steepness of the response was detected, resulting in a lower total response with 3 Gy dose. As HbT was calculated as a sum of these two, it shows a steeper response but smaller total response with 3 Gy dose. In the water signal, no difference could be detected between the doses.

**Fig. 5:**
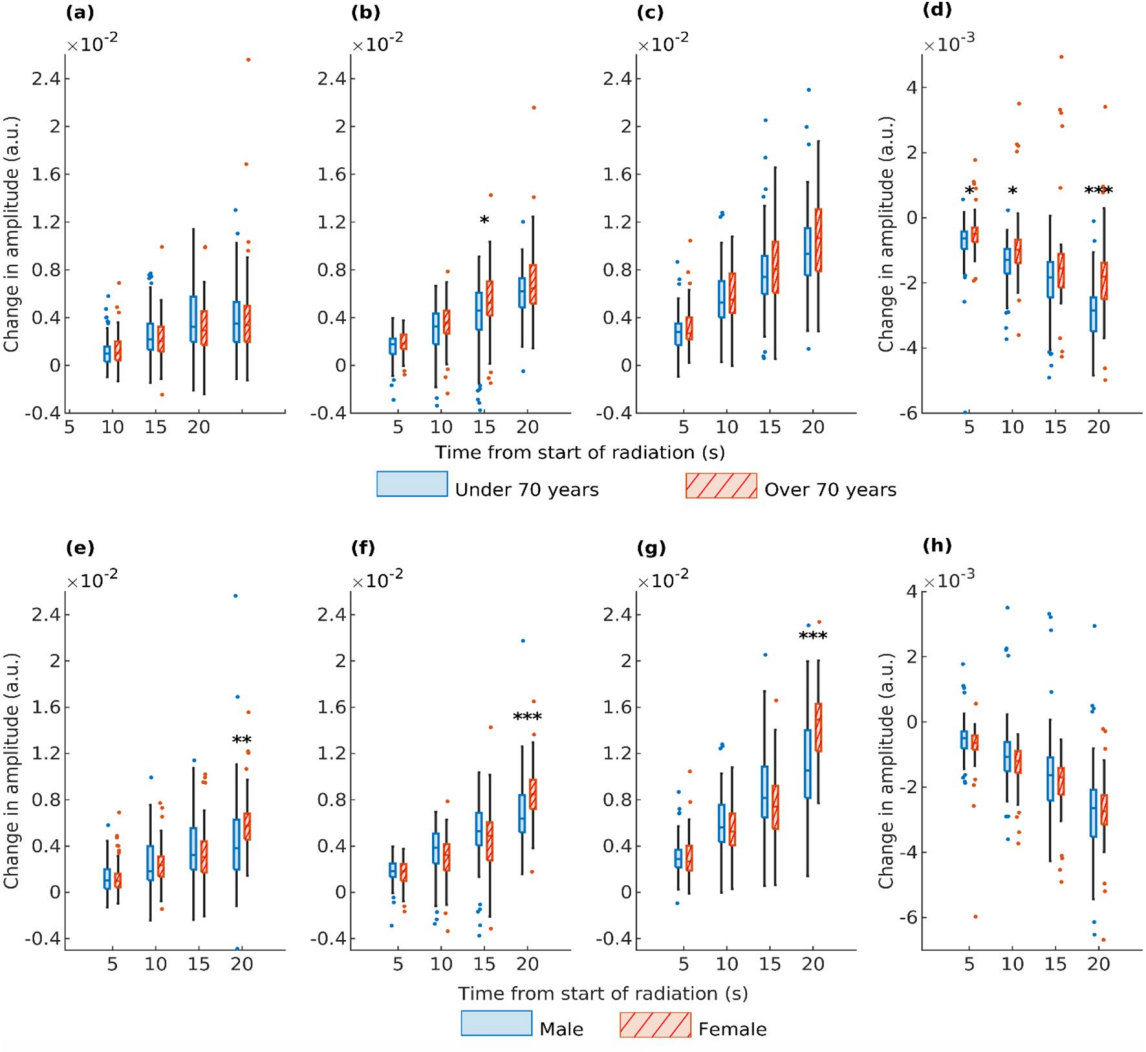
The figure illustrates the change in the low-pass filtered amplitude of HbO (**a**, **e**), HbR (**b**, **f**), HbT (**c**, **g**), and water (**d**, **h**) signals between the start of irradiation and 5, 10, 15, and 20 s after initiation. In panels (**a**–**d**), significant *P* values (*P < .05, **P < .01, and ***P < .001) indicate the differences in signal amplitude change between patients under 70 years old (blue) and those over 70 years old (red). In panels (**e**–**h**), the comparison is between male (blue) and female (red) patients. There were 17 patients (measurements *n* = 58) under 70 years old and 14 patients (*n* = 48) over 70 years old, and 16 female patients (*n* = 57) and 15 male patients (*n* = 50). *P* values reveal a more significant change in the water signal for patients under 70 years old compared to those over 70 years old. Between male and female patients, a larger response is observed in the change of HbO, HbR, and HbT signals 20 s after the start of irradiation in female patients

Our results also show slight differences in response to irradiation between different genders and different aged patients (see Fig. 5). Between patients over and under 70 years old, only statistically significant difference can be seen in the water signal, which shows a stronger response in younger patients. Between male and female patients, our results show a larger total response in HbO, HbR, and HbT signals for females.

## Discussion

In the current study, the immediate cerebral hemodynamic and water content response to WBRT was recorded. Our results show a statistically significant increase in HbO, HbR and consequentially in HbT, signifying increased blood volume and a decrease in water content as soon as 5 s from the start of irradiation. As fNIRS has limited measuring depth, our results come from a limited brain volume within the brain cortex on patients’ forehead [19].

We conclude our signal to show immediate irradiation-induced BBBD. NIRS has been previously used to monitor BBBD in humans after intra-arterial mannitol infusion by Kiviniemi et al. [18]. Their findings revealed a notable decline in HbO and HbR signals after mannitol infusion, a recognized blood–brain barrier-opening agent. This observed pattern closely resembled our study, where both signals exhibited a subsequent elevation, leading to HbO surpassing the original baseline and maintaining an elevated state, while HbR initially increased to the original baseline level but later declined below it. The irradiation induced increase in HbO and HbR signals show similar response, without the initial drop as there is no mannitol infusion, and our recording was too short to assess whether the rise in HbO and HbR signals persists. Another study by Myllylä et al. [21] used fNIRS to record BBBD induced with mannitol and focused ultrasound (FUS) in mice. Their fNIRS signal response to FUS during the first 20 s shows an increase in HbO and HbR and a decrease in water, like the response recorded in the current study during irradiation. The increase in HbO and HbR signal indicates an increased blood volume within the recorded area. NIRS studies are not the only studies linking increased blood volume with BBBD; the connection was also done by Zhang et al. [22], who studied the association between changes in cerebral blood flow and BBB permeability in a region of spontaneous intracerebral hemorrhage using computed tomography. They linked increased cerebral blood volume and decreased blood flow with BBBD.

Studies have well-documented radiotherapy-induced BBBD, though the precise onset time remains unclear [23–27]. Yuan et al. [23] measured BBB permeability on mice receiving 40 Gy of whole brain irradiation in 2 Gy daily doses. They observed a significant change in BBB permeability 90 days after the fractionated radiotherapy, but not during it. Cao et al. [26] monitored BBB permeability and blood plasma volume in patients treated with partial brain radiotherapy during and after the radiotherapy. They saw a significant increase in blood plasma volume and BBB permeability in six weeks from the start of treatment, in tissue exposed to a total radiation dose of at least 20 Gy. With a greater single dose, a significant increase in BBB permeability has been observed already in 2 h after irradiation in rats, but in the same study no significant change in hemodynamic conditions was recorded [25]. Results of the current study suggest that BBBD happens immediately upon irradiation. That would have been impossible to detect in previous studies, where BBB permeability was accessed between or after irradiations.

BBBD is known to cause an increase in the total brain water content in the form of vasogenic edema [27–29]. This does not contradict with the recorded decrease of water signal during irradiation in the current study, as our water signal comes from a limited brain volume. The water signal is affected by both water bound with blood and free water, which contains both intracellular and extracellular water. Cerebrospinal fluid (CSF) is a major component in the signal. It has also been demonstrated to be the primary source of early edema fluid after an ischemic stroke in mice by Mestre et al. [30]. The decrease in our water signal can signify the movement of CSF since it is well known for radiotherapy to induce edema in the brain [11–13, 31].

We observed a difference in the immediate response to irradiation between patients with different treatment dose fractionations. Fractionation of five 4 Gy fractions is the standard fractionation used for WBRT in the hospital, and fractionation of ten 3 Gy fractions is only used when an oncologist considers it to be less taxing on the patient. However, there are no clear criteria for the division; hence fractionation depends heavily on the oncologist. Therefore, no clear cause for the recorded difference could be found. It is worth noting that the 3 Gy dose group consisted of only three patients leaving the sample size too small for any definite conclusions. Considering the limitations of the current study and that no difference in the overall survival or symptom control has been proven between these two fractionation schedules previously [32], more studies are needed to assess the cause and biological significance of the recorded difference.

In the current study, differences in the response to irradiation were noted among patients based on age (specifically, those under and above 70 years) and gender. In older patients, a smaller decrease in the water signal was recorded during irradiation, while female patients had a greater total increase in HbO, HbR, and HbT signals than male patients. These kinds of differences in immediate response have not been studied before. However, differences in the age and gender groups have been shown following the radiotherapy. For example, hippocampal BBB permeability following partial brain radiotherapy has been previously studied by Farjam et al. [33]. One month after radiotherapy, they detected an increase in BBB permeability that significantly depended on gender and age. In patients who received a total dose over 19 Gy, there was a greater increase in BBB permeability in female patients and patients over 50 years old. Moreover, in retrospective studies including patients with brain metastases treated with brain radiotherapy, younger age and female sex have been connected to higher survival rates [34–36]. While younger patients are shown to have a longer overall survival, the difference is at least partly caused by other factors, such as comorbidities [37–39]. The aging process is associated with physiological changes that reduce toleration to radiation, predisposing older patients to vascular radiation damage [37] which may affect the signal seen in the study. Effect of gender in radiotherapy and cancer development have been shown in multiple studies reviewed by De Courcy et al. [40] and Li et al. [41]. Differences in treatment response are linked to greater radiosensitivity in women, hormonal signaling, and neuroprotective properties of estrogen [40, 41]. Source of the differences seen in this study and their clinical implications should be assessed in future studies.

There are a few limitations related to the current study. Firstly, as FIMRT was used for some, but not with all patients, there was no irradiation at the time points of 15 and 20 s in all measurements. Secondly, as mentioned before, we had a limited sample size. The third limitation was that in long-term use, irradiation of the optical fiber tip started to slowly decrease its light transparency especially below 800 nm, affecting the detected signal quality for the 690 nm wavelength. Importantly, irradiation did not cause immediate effects to the detected light signal which was ensured by several test measurement using phantoms. Due to variations in the timing of radiation exposure among patients, comparing immediate effects with slightly delayed ones is inevitable. This adds uncertainty to comparisons at the end of the irradiation (last ~ 4 s), partly limiting interpretation of results. Thus, the difference seen between patients of different genders could arise from an immediate or a slightly delayed response. The limited sample size may result in the study being underpowered to detect all individual differences in the irradiation response and limit generalizability of our findings. Nevertheless, the lack of prior studies assessing both immediate and delayed irradiation responses on a seconds-scale timeframe, coupled with the consideration of individual differences among patients, necessitates a comprehensive evaluation of these potential limitations in future research. The condition of optical fibers affects the quality of measurements, and due to the novelty of the method, their irradiation-induced attenuation was not considered from the start. Although this limitation was quickly minimized by recording the condition of optical fibers and replacing them as necessary, some channels still produced poor-quality data that had to be excluded during analysis, while others exhibited increased noise as the fibers deteriorated. While this added noise did not impact the overall trends across recordings, it limits the strength of conclusions that can be drawn at the individual level.

Future research should aim to address these limitations and build upon the current findings. Minimizing variability in factors such as the condition of optical fibers, the distance between source and detector fibers on the patient’s forehead, and differences in treatment protocols could enhance the accuracy of studying individual differences in irradiation response. Standardizing these variables will allow for a more reliable assessment of how patient-specific irradiation responses can inform personalized treatment planning and improve the prediction of clinical outcomes. Further studies are also needed to explore the immediate effects of irradiation-induced blood–brain barrier disruption (BBBD). A deeper investigation into the immediate and delayed BBBD responses could validate the conclusions of this study and offer new insights into optimizing therapeutic strategies. Additionally, exploring larger and more diverse patient cohorts would help to improve the statistical power of future studies, thereby enhancing the reliability and generalizability of the results.

As radiotherapy remains the primary treatment method for brain metastases, it is vital to understand the immediate effects it causes in the brain. This study, which measured cerebral hemodynamics and water content during irradiation, revealed an immediate response that can be effectively captured using fNIRS. The observed immediate increase in total hemoglobin and decrease in cerebral water within the measured area suggest that irradiation induces immediate BBBD. The response showed age, gender, and dose-dependent differences between patients, highlighting the potential of our methodology for individually tailored radiotherapy and more effective chemotherapy.

## Supplementary Information

Below is the link to the electronic supplementary material.

Supplementary file1 (PDF 435 KB)

## Data Availability

A fully anonymized data set can be made available upon request.
