# Supplementary material for: Immediate Irradiation Induced Cerebral Water and Hemodynamic Response in Whole Brain Radiotherapy
Source: Ann Biomed Eng. 2024 Dec 4;53(3):673–82. doi: 10.1007/s10439-024-03663-1 (PMC11836184; doi:10.1007/s10439-024-03663-1)
Supplement: Supplementary file 1 — Supplementary file1 (PDF 435 KB) [file 10439_2024_3663_MOESM1_ESM.pdf]

Supplemental Information (SI) to:
Immediate irradiation induced cerebral water and hemodynamic response in whole brain radiotherapy

Heli Miettinen [a,*], Jesse Lohela [b,c], Sadegh Moradi[c,e], Kalle Inget [c,d], Juha Nikkinen [b,c,d], Teemu Myllylä [c,e], Sakari S. Karhula [b,c,d], Vesa Korhonen [a,c,d]

[a]Oulu University Hospital, Department of Diagnostic Radiology, Oulu, Finland
[b]Oulu University Hospital, Department of Oncology and Radiotherapy, Oulu, Finland
[c]University of Oulu, Research Unit of Health Sciences and Technology, Oulu, Finland
[d]Medical Research Center, Oulu, Finland
[e]University of Oulu, Optoelectronics and Measurement Techniques Unit, Oulu, Finland
*Address correspondence to Heli Miettinen, heli.a.miettinen@pohde.fi

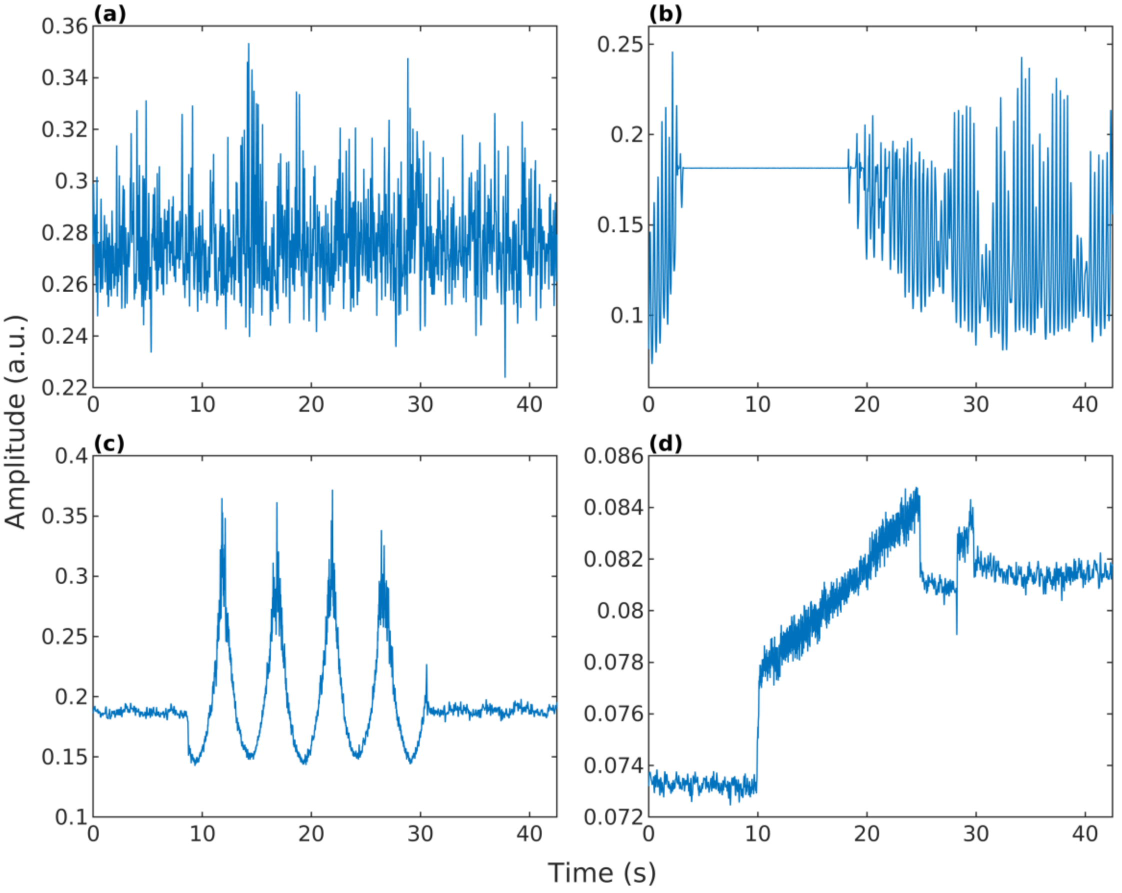

Fig. S1 HbR signal of few rejected channels during section of interest as an example of types of rejected signals. (**a**) shows a signal rejected due to SNR being less than one, while (**b**), (**c**) and (**d**) show types of artefacts that resulted in rejection of the channel.
